# Case report: Resolution of Guillain-Barré syndrome in a patient with dual primary tumors after treatment with rituximab

**DOI:** 10.3389/fneur.2024.1348304

**Published:** 2024-02-21

**Authors:** Desheng Zhang, You Wang, Fuxiang Zhou

**Affiliations:** Department of Radiation and Medical Oncology, Zhongnan Hospital of Wuhan University, Wuhan, China

**Keywords:** Guillain-Barré syndrome, immune checkpoint inhibitors, tumor, targeted therapy, case report

## Abstract

Guillain-Barré syndrome (GBS) is a rare immune-related adverse event (irAE) that can occur in solid tumors such as hepatocellular carcinoma, gastric cancer, breast cancer, and colorectal cancer. It is characterized by progressive myasthenia and mild sensory abnormalities. The emergence of immune checkpoint inhibitors (ICIs) has significantly improved cancer patients’ life expectancy but can also trigger various irAEs, including GBS. We report a rare case of GBS in a 64-year-old male patient with dual primary tumors of the colon and stomach who received toripalimab and chemotherapy for liver metastases. After five treatments, the patient experienced weakness and numbness in his limbs. Lumbar puncture, electromyography, and other tests confirmed the diagnosis of GBS. Intravenous immunoglobulin (IVIG) and methylprednisolone did not improve the patient’s symptoms, but rituximab, which is not a standard regimen for GBS, was effective in eliminating B cells and improving symptoms. Following this, we effectively shifted from a regimen combining immunotherapy and chemotherapy to a targeted therapy regimen, resulting in prolonged patient survival. Currently, limited studies have been undertaken to evaluate the efficacy of rituximab in managing refractory neurological adverse events associated with ICI therapy. Using this case, we reviewed similar cases and formed our views.

## 1 Introduction

GBS is an autoimmune-mediated peripheral neuropathy characterized by peripheral neuropathy, weakness, cranial nerve damage, and elevated cerebrospinal fluid protein levels (1). While GBS is commonly caused by infections such as cytomegalovirus, EBV, or *Campylobacter jejuni*, it has also been observed in a small number of cancer patients. Studies have indicated higher morbidity and mortality rates in patients with gastric adenocarcinoma, hepatocellular carcinoma, colon cancer, and Hodgkin’s lymphoma (2). The use of platinum or ICIs can lead to the development of GBS in cancer patients (3, 4). In non-tumor individuals, the prognosis of GBS is favorable, with 80% experiencing complete recovery and a 5% mortality rate (5). Intravenous immunoglobulin and plasma exchange may improve prognoses. For cancer patients, traditional treatment for GBS may be ineffective, as evidenced by a mortality rate of 22% (6). For such patients unresponsive to standard therapies, there is a growing need for more precise treatments, such as rituximab, a B-cell depleting monoclonal antibody. Although definitive studies supporting its routine use for GBS treatment are lacking, multiple cases of refractory GBS have demonstrated significant improvement in functional outcomes following rituximab administration (7, 8).

## 2 Case presentation

An elderly patient presented at the hospital with severe limb weakness. The patient had dual primary tumors in the colon and stomach and had undergone six cycles of FOLFOX chemotherapy and eight cycles of FOLFOX4 chemotherapy. The patient was diagnosed with liver metastases from gastric cancer, confirmed by immunohistochemistry. The patient received four cycles of chemotherapy with albumin-bound paclitaxel and platinum, along with five cycles of toripalimab immunotherapy. However, just one week following the fifth cycle of immunotherapy, the patient was hospitalized due to significant weakness of limbs. Electromyography (EMG) was conducted, revealing peripheral nerve damage involving motor and sensory fibers, as well as proximal nerve roots. The patient’s extremities’ motor and sensory nerve conduction studies are shown in Table 1. A lumbar puncture showed albumin cytologic dissociation, and positive anti-sulfatide antibodies were detected in the patient’s serum and cerebrospinal fluid. Based on the evidence, the patient was diagnosed with GBS.

**Table 1:** The result of electromyography examination.

Nerve	Site	Latency (ms)	Amplitude (mV)	Conduction velocity (m/s)	Distance (mm)
MNCS					
Left ulnar	Wrist	2.67	8.3		50
	Below elbow	7.39	7.40	48.7	230
	Above elbow	9.42	7.00	49.3	100
Left median	Wrist	3.33	5.00		40
	Elbow	8.04	5.10	52.0	245
Left tibial	Ankle	4.33	5.6		95
Right tibial	Ankle	4.25	5.20		85
Left peroneal	Ankle	3.88	1.87		50
	Bl Fib.head	12.6	1.90	37.3	325
Right peroneal	Ankle	4.17	2.10		30
	Bl Fib.head	13.10	2.50	35.30	315
SNCS					
Left ulnar	Wrist	2.46	18.20	48.80	120
Left median	Wrist	2.44	19.00	53.30	130
Left superficial peroneal	Lower leg	1.97	6.70	48.20	95.0
Right superficial peroneal	Lower leg	1.96	6.70	43.40	85
Left sural	Middle leg	2.27	7.50	39.6	90
Right sural	Middle leg	2.48	9.90	44.4	110

MNCS, motor nerve conduction studies; SNCS, sensory nerve conduction studies; Bl Fib. head, below fibula head.

Following the diagnosis, the patient received treatment with intravenous methylprednisolone at a dose of 500 mg and immunoglobulin (IVIG) at a dose of 20 g (0.4 g/kg) per day, following the Chinese Society of Clinical Oncology (CSCO) Guidelines for managing immune checkpoint inhibitor-related toxicity in 2021. Unfortunately, this treatment did not yield significant results. Consequently, the patient underwent two cycles of rituximab treatment to eliminate B cells. Before treatment, the patient presented with grade 3 upper limb muscle strength, grade 1 lower limb muscle strength, absence of patellar reflex (−), and absence of Babinski sign (−). Following treatment, the patient’s muscle strength improved to grade 4 in the upper limbs and grade 2 in the lower limbs. The patient’s muscle weakness of the lower limbs also improved at day 7 from 1/5 to 2/5, according to the Medical Research Council (MRC) Scale. This led to symptom relief and the restoration of independent leg movement, although walking was still not possible. To address GBS, we temporarily paused anti-tumor therapy, which resulted in rapid tumor progression and a dramatic increase in the patient’s tumor marker (CA199) to over 4,000 U/mL. Subsequently, we discontinued toripalimab and initiated targeted therapy with bevacizumab along with chemotherapy using oral Capecitabine. Genetic testing revealed an ARID1A mutation in our patient, leading us to administer everolimus targeted therapy following National Comprehensive Cancer Network (NCCN) guidelines. Post-treatment, the patient’s CA199 level decreased to 2000 U/mL. We conducted a review of the liver MRI, revealing that the metastases had diminished in size compared to previous scans, suggesting the treatment’s efficacy. The patient then returned home to recover, regrettably, he succumbed in intensive care 8 months later due to severe gastrointestinal bleeding caused by a tumor.

## 3 Discussion

To our knowledge, this report represents the first identification of GBS in a patient with two primary tumors. It is important to highlight that the selected antineoplastic regimen included both chemotherapy and immunotherapy. Therefore, it remains uncertain whether the patient’s GBS was triggered by the chemotherapy or the immunotherapy. Both types of drugs have the potential to induce GBS (9). In similar instances, the patients of Ding et al. (10) and Manam et al. (11) were also treated with a combination of chemotherapy and ICIs. Both authors considered GBS as a possible result of ICIs. Ding cited the limited references on chemotherapy induced GBS and the short duration of illness in her patients as factors. That argument lacks persuasiveness. Apart from GBS, antineoplastic chemotherapeutic agents can directly induce neurotoxicity and lead to chemotherapy-induced peripheral neuropathy (CIPN). It has been demonstrated that immune checkpoint inhibitors are associated with a higher risk of neurological adverse events compared to control groups, yet this risk is significantly lower when compared to chemotherapeutic agents (10, 12). Both conditions have comparable clinical symptoms, which may lead to weakness and numbness in the limbs, as well as sensory abnormalities. They can be differentiated through the analysis of cerebrospinal fluid and anti-ganglioside antibodies. After confirming a diagnosis of GBS and ruling out CIPN, consideration should be given to ICIs as the potential cause. In 2006, the patient underwent six FOLFOX chemotherapy cycles to treat his colorectal cancer. However, the individual did not exhibit comparable symptoms during that period. Based on the patient’s medical history, medication usage, clinical manifestations, and laboratory findings, we conclude that Toripalimab is the most likely cause of GBS. Although we suspect that the immune checkpoint inhibitors (ICIs) are the culprit for inducing GBS in our patient, we cannot disregard the impact of chemotherapy drugs. Prior to their onset of lower limb weakness, the patient experienced significant myelosuppression. Chemotherapy drugs can contribute to this phenomenon, while a weakened immune system may facilitate the induction of GBS by ICIs. That is the reason why the combination of chemotherapy and immunotherapy is more liable to cause GBS in contrast to immunotherapy by itself.

Due to the unclear pathogenesis of GBS and the intricate clinical symptoms, there currently exists no proven and efficient treatment for GBS. According to the 2021 CSCO guidelines for ICIs-related toxicity, both methylprednisolone and IVIG are established therapies for GBS (10). ICI-induced GBS is believed to be mainly linked to T-cell activation (13). While the exact mechanism of action of IVIg in GBS is unknown, it has also been suggested that it may be related to the provision of anti-specific antibodies that regulate the expression and function of the Fc receptor (14). Some studies have shown that, mere IVIg treatment might have limited therapeutic efficacy; thus, it is usually combined with high doses of systemic corticosteroids. This combination therapy seems to more effectively target the underlying immunological mechanisms involved (3, 15). Furthermore, in cases of refractory Guillain-Barré syndrome, where conventional treatment regimens may not produce the desired outcomes, alternative immunosuppressants such as TNFα inhibitors, mescaline or anti-thymocyte globulin, and monoclonal antibodies like rituximab, represent viable therapeutic options (7, 8, 10).

Rituximab is an anti-CD20 monoclonal antibody that leads to B cell depletion through complement-dependent cytotoxicity (CDC), antibody-dependent cell cytotoxicity (ADCC) involving NK cells, and phagocytosis by macrophages and neutrophils (16). In an effort to better understand the role of rituximab in Guillain-Barre Syndrome, we have summarized a review of similar cases (refer to Table 2). In the cases we examined, rituximab has demonstrated a favorable impact on refractory Guillain-Barre syndrome. Rituximab induces B-cell depletion by targeting CD20 on the surface of B-cells. While it does not directly affect T-cells, it also promotes recovery by eliminating potential pathological antibodies and/or inhibiting harmful pro-inflammatory effects (18, 20). Furthermore, there is a hypothesis that ICI-induced GBS may be linked to decreased tolerance to ganglioside-associated epitopes, as well as uncontrolled peripheral immune responses resulting from disrupted function of normal immune checkpoint molecules (21). Research has demonstrated that rituximab plays a significant beneficial role in improving the antibody-dependent subtype of GBS (AMAN) (8). While the specific mechanism still needs further study.

**Table 2:** Summary of the reported cases with rituximab treatment for GBS.

References	Age	Gender	Causes of GBS	Clinical features	GBS therapy	Outcome
Gu et al. (17)	49	Female	Ipilimumab 3 mg/kg Nivolumab 1 mg/kg	Weakness and pain of the 4 limbs Sensory loss	(1) IVIg and steroids (2) Steroids, plasma exchange, mycophenolate, and rituximab	Significant improvement in nerve and muscle strength
Janssen et al. (3)	74	Male	Ipilimumab 10 mg/kg q3w	Tetraparesis Distal hypoesthesia of the 4 limbs Diffuse areflexia	(1) Steroids (2) IVIg and Infliximab (3) Plasmapheresis (4) IVIg and rituximab	Fatal due to respiratory failure/pneumonia
Beshir et al. (18)	69	Male	Systemic lupus erythematosus	Severe generalized weakness diplopia and swallowing difficulty	(1) IVIg and steroids (2) rituximab plasma exchange	No motor deficit Intermittent paraesthesia of the 4 limbs
Reddy (19)	9	Female	Systemic lupus erythematosus	Weakness and pain of the 4 limbs Sensory loss	(1) IVIg and steroids (2) rituximab	Full recovery
Ostronoff et al. (8)	58	Male	Allogeneic hematopoietic SCT	Weakness and pain of the 4 limbs Facial palsy	(1) IVIg (2) rituximab	Significant muscle strength recovery

In cases where a patient is considered cured or requires ongoing antineoplastic drug treatment due to tumor progression, it is not advisable to continue with the initial treatment regimen. Doing so may lead to a potential reoccurrence of GBS. In our specific case, given the rapid progression of the patient’s tumor, we opted for a combination of targeted therapy and oral chemotherapy, which yielded remarkable results. Despite the patient’s eventual passing due to gastrointestinal bleeding, our treatment contributed to extending his survival to some extent, granting patient to live an additional 8 months. If a patient necessitates ICIs, it might be beneficial to consider substituting the PD-1 inhibitor with a PD-L1 inhibitor. Some studies have suggested that PD-L1 inhibitors may lead to fewer adverse events compared to PD-1 inhibitors.

## 4 Conclusion

This marks the initial report documenting the association between Toripalimab and GBS. GBS is a rare condition, particularly among patients with tumors. IVIg and methylprednisolone stand as the preferred treatments. However, if these treatments prove ineffective, exploring the potential co-administration of certain non-traditional drugs becomes crucial. Early detection and prompt treatment hold paramount im-portance for cancer patients, as time plays a critical role in their survival. Our case report offers valuable insights into effective treatment options for ICIs-induced GBS.

## Data availability statement

The original contributions presented in the study are included in the article/supplementary material, further inquiries can be directed to the corresponding authors.

## Ethics statement

Written informed consent was obtained from the individual(s) for the publication of any potentially identifiable images or data included in this article.

## Author contributions

DZ: Writing – original draft. YW: Writing – review & editing. FZ: Supervision, Writing – review & editing.
